# St. Louis Clinic

**Published:** 1893-05

**Authors:** Z. F. Lillard

**Affiliations:** Reporter; Tyler, Texas


### ST. LOUIS CLINIC.

[Reported by Dr. Z. F. Lillard, of Tyler, Texas.]

### TWO SURGICAL CASES.

CASE I. Sam Williams, colored, age 22 years, was admitted to the hospital at 6:35 p. m. Friday, March 17th.

An examination revealed a gun shot wound, the ball having entered the neck on right side an inch below the external auditory canal. He was unconscious, his breathing labored. The tissues below the jaw on the right side were disturbed greatly by what was diagnosed as a hæmatoma. This pressed upon the larynx and was considered the sole cause of the disturbance of respiration until the post mortem gave rise to the question as to whether nerve fibres severed aided in producing such an effect. Tracheotomy was an immediate necessity, and was performed by Dr. Marks the superintendent of the hospital. But for the operation he could not live five minutes it seemed. A tube was inserted, his neck dressed antiseptically and patient put to bed.

An intelligent attendant was at the bedside with orders not to leave the patient. This attendant states that soon after he took charge of the case he noticed paralysis of left side of face, arm, and leg, a symptom not discovered till next day by the surgeons. He was conscious a portion of the time Saturday and Sunday but his hemiplegia was permanent. He remained in this condition till death, March 20, at 8:45 p. m.

POST MORTEM.—A probe was inserted along track of bullet till it could be felt on the opposite side of neck. An incision was next made along the upper portion of the right common carotid which was dissected out. The external branch was next followed up and found intact. Next the internal carotid was followed up and found to be severed about half in two. There had been no hemorrhage from the hole of entrance of the ball but the blood from the internal carotid had infiltrated the tissues below the inferior maxilla on right side. Behind the œsophagus and extending over a portion of the left side the blood had formed more a hæmatoma. This had undoubtedly constricted the lyrnx and helped to make an immediate tracheotomy a necessity. The dissection was not fine enough to determine the damage to the nerves. The ball also struck a vertebra and shattered off a small piece or two.

Cranium next opened, and brain removed. Left half of brain was of normal firmness, and otherwise normal in its entirety. Right half softer than normal, and in two or three places completely degenerated.

Other organs not examined. The interference in the circulation explains the paralysis, the unconsciousness, and the succeeding brain degeneration.

The readers of the JOURNAL can see that the paralysis at least would perplex most observers.

CASE 2. Caroline, age 46, colored. February 28, 9 o'clock p. m., came into hospital with a history of having been shot an hour or two previously. The ball had entered left eye, and made its exit immediately above the right ear, almost grazing the penna. Was conscious, and very talkative from the influence of liquor. The head was shaved and parts made as clean as possible. The left eye enucleated, and a horse-shoe shaped incision, convexity upward, was made, and flap dissected up, exposing the hole of exit. This hole was enlarged with bone forceps, from which a small portion of cerebral tissue exuded. The introduction of the finger revealed the fact that the optic nerve to the right eye had been severed. A drainage tube was inserted, and antiseptic dressing applied. As usual, this dressing was not disturbed till her temperature showed that the wound was not doing well. Reference to her chart shows that her temperature reached 101 on the evening of March 1st, 102 on the evening of the 5th, and 106 on the morning of the 15th, with lower temperature between the above dates. It was normal on the mornings of the 11th and 13th, with higher temperature on all other mornings.

Wound was examined about the fifth day after operation, and thought to be doing well. About the tenth day examined again, some of the stitches removed, one or two stitch abscesses discovered, and a few drops of pus pressed out of hole of exit of the bullet, and bichloride gauze pushed in the opening at lower and posterior end of incision to establish drainage.

On the morning of the 15th, when fever reached 106, dressing was again removed. There was much œdema on right side of head and face, and considerable pus on dressing. By enlarging the opening of exit and making an incision at upper and anterior portion of the flap, more pus was evacuated. From this time on wound has been dressed daily, and gauze inserted to secure drainage. Temperature has gradually receded. Discharges were at one time very offensive, but after the use of chloride of zinc, 8% solution, and bichloride solution 1 to 1000, the wound has been almost odorless. Chances of recovery, at present, are good. Of course the sight of both eyes is lost, but patient insists that if she had her "specs" she could see.

